# Hydrocephalic Parkinsonism: lessons from normal pressure hydrocephalus mimics

**DOI:** 10.1186/2054-7072-1-2

**Published:** 2014-10-29

**Authors:** Brian W Starr, Matthew C Hagen, Alberto J Espay

**Affiliations:** Gardner Family Center for Parkinson’s Disease and Movement Disorders, Department of Neurology, University of Cincinnati, 260 Stetson St, Suite 2300, Cincinnati, OH 45267-0525 USA; Department of Pathology, Division of Neuropathology, University of Cincinnati, Cincinnati, OH USA

**Keywords:** Normal pressure hydrocephalus, Ventriculomegaly, Progressive supranuclear palsy, Dementia with Lewy bodies

## Abstract

**Background:**

Hydrocephalus is an under-recognized presentation of progressive supranuclear palsy (PSP) and dementia with Lewy bodies (DLB).

**Methods:**

We describe four normal pressure hydrocephalus (NPH)-like presentations of pathology-proven PSP (n = 3) and DLB (n = 1) and review the literature on the hydrocephalic presentation of these atypical parkinsonisms.

**Results:**

Despite the presence of ventriculomegaly disproportionate to the extent of parenchymal atrophy, all patients demonstrated early postural impairment and/or oculomotor abnormalities that encouraged a diagnostic revision. Hallucinations were the only early atypical manifestation of the hydrocephalic DLB presentation.

**Conclusions:**

Early postural impairment, falls, oculomotor impairment, and/or hallucinations are inconsistent with the diagnosis of NPH and suggest PSP or DLB as the underlying NPH mimic. We postulate that previously reported cases of “dual” pathology (e.g., NPH *and* PSP) actually represent the hydrocephalic presentation of selected neurodegenerative disorders.

**Electronic supplementary material:**

The online version of this article (doi:10.1186/2054-7072-1-2) contains supplementary material, which is available to authorized users.

## Background

Hydrocephalus as an imaging finding is commonly interpreted as potentially representing normal pressure hydrocephalus (NPH) largely because of the favorable therapeutic implications [1]. However, NPH is a relatively rare disorder, recently calculated to be 1.19/100,000/year, and reduced further to 0.36/100,000/year when defined as *sustained* improvement at 3 years after ventriculoperitoneal shunt (VPS) placement [1]. Between 1995 and 2003, from 411 patients referred as NPH to the Mayo Clinic, only 41 were tentatively endorsed as such and barely 14 experienced gait improvement after a trial of cerebrospinal fluid removal, the *sine qua non* for the diagnosis. Of the 12 patients who underwent VPS placement, definite gait improvement was present in only 6 by one year and just 4 after 3 years, one third of the original VPS-treated cohort. Remarkably, pathology available in 5 patients in the VPS-treated cohort revealed the presence of neurodegenerative disorders, progressive supranuclear palsy (PSP) and dementia with Lewy bodies (DLB), each accounting for a case.

Because of the large volume of NPH referrals to tertiary care centers, predicting a *sustained* response to VPS placement is paramount to justifying this invasive procedure. We examined four patients referred to us for suspected NPH but whose pathology demonstrated PSP and DLB. The preliminary diagnosis of NPH in these patients was based on the interpretation of imaging features, namely the presence of hydrocephalus judged disproportionate to the extent of surrounding parenchymal atrophy. While a careful neurological examination suggested further NPH work up was unwarranted in three patients, cerebrospinal fluid diversion in one (case 4) supported this diagnosis by yielding subjective benefits in gait and urinary function. In addition to early impairment postural reflexes, other clinical red flags against NPH emerged between 12 and 24 months. We suggest that PSP and DLB, as previously reported for Alzheimer disease (AD), is capable of generating a neuroimaging profile suggestive of communicating hydrocephalus, with ventriculomegaly disproportionate to the extent of parenchymal atrophy, prompting a diagnostic consideration for NPH and potentially misdirecting treatment toward cerebrospinal fluid diversion.

## Methods

### Case reports

Four consecutive patients referred to our center for suspected NPH for whom brain autopsy was obtained were identified in our center from the years 2007–2012. After evaluation in our center, VPS placement was discouraged for the first three patients but pursued in the fourth to no avail. Written informed consent was obtained from all patients for the publication of patient-related data and videotapes.

## Results

### Case 1

**Figure 1:**
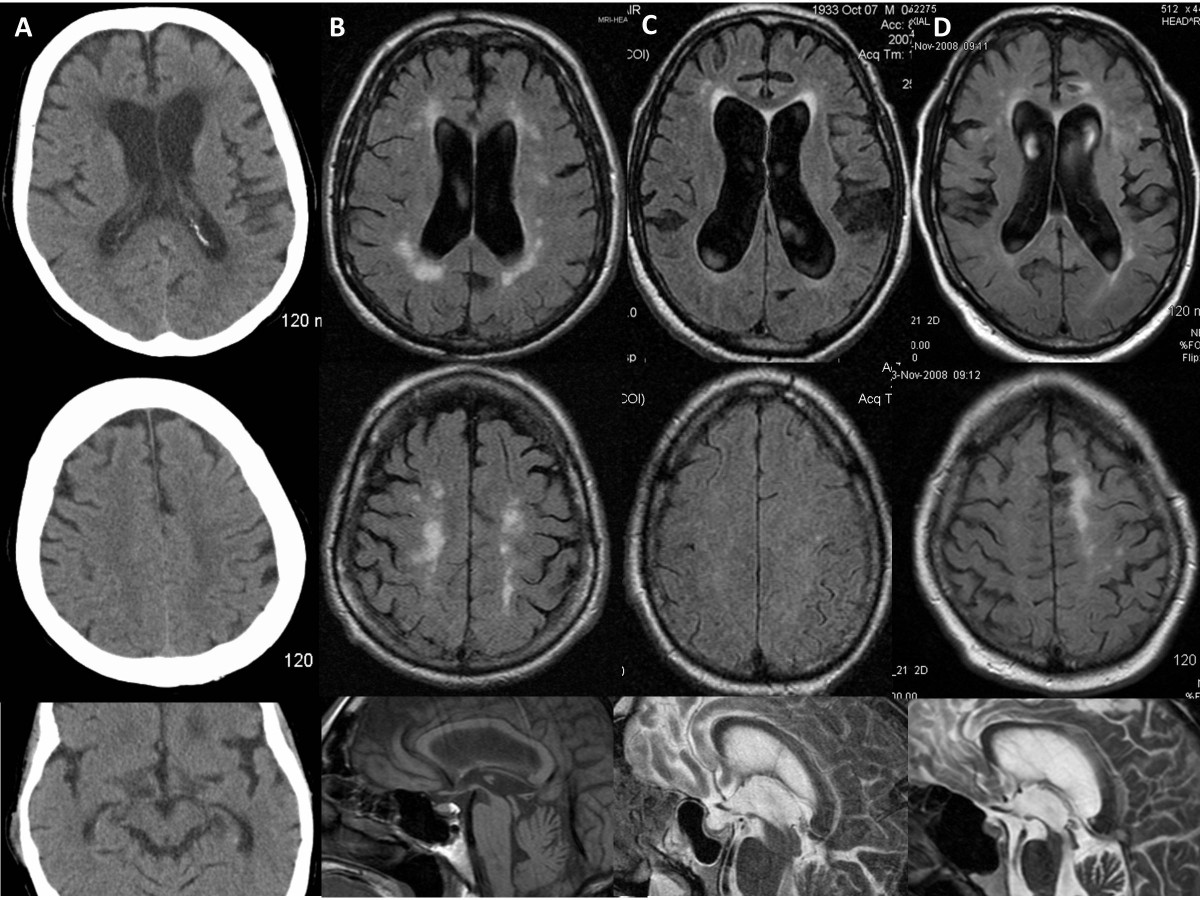
**Neuroimaging of the NPH suspected cases.** Head CT **(A)** and FLAIR sequences of brain MRI **(B-D)** for cases 1–4 (A-D) showed mild to moderate ventriculomegaly (upper row), with relatively mild or absent (Case 3) associated parenchymal atrophy as judged by the apical axial cuts (middle row). Some degree of midbrain atrophy is appreciable in all cases, including the one ultimately diagnosed as DLB on pathology **(C)** despite a clinical picture that suggested a rapidly progressive form of PSP. Leukoencephalomalacia in the right cerebellar hemisphere and left parasagittal frontal lobe was due to remote stroke in Case 4.

**Figure 2:**
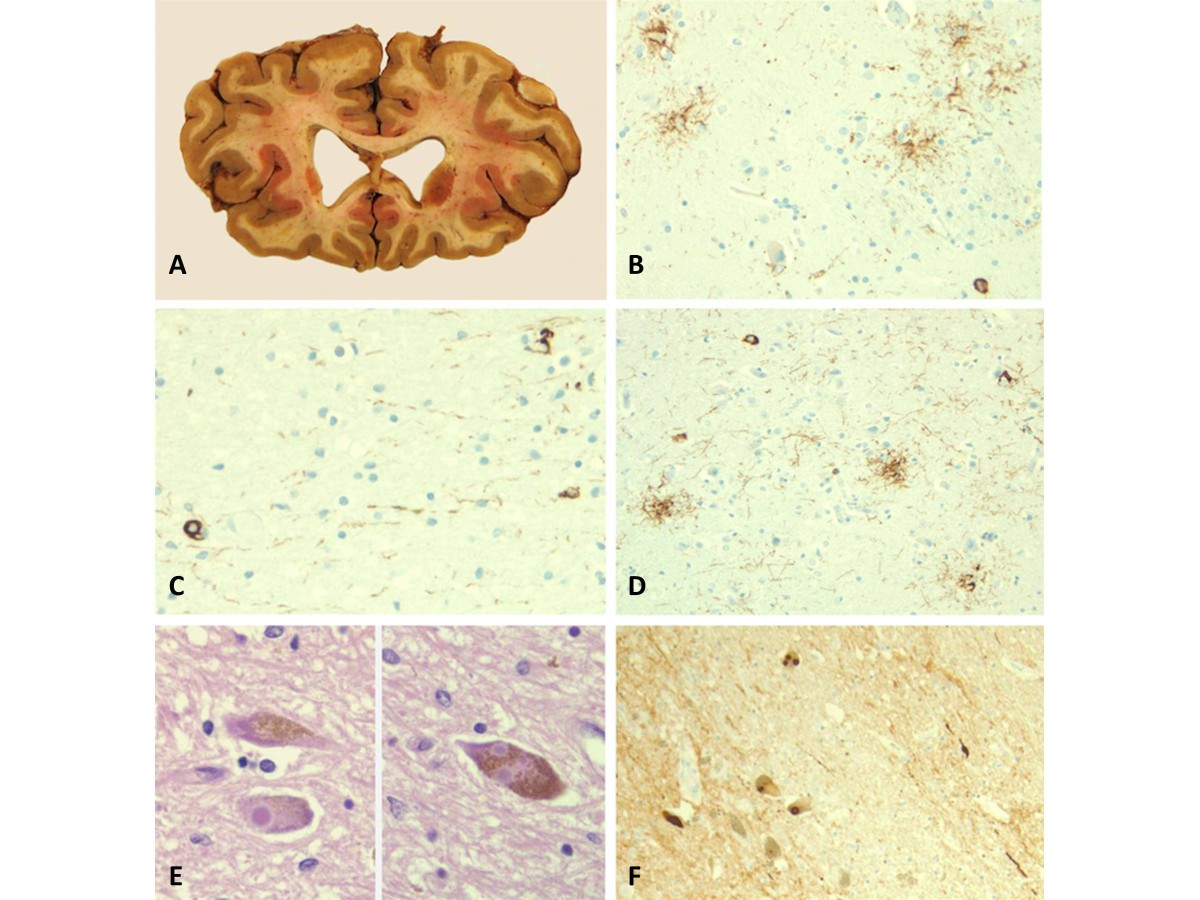
**Neuropathology images of selected cases. A**. (Case 1) Coronal section demonstrating enlargement of the anterior horns of the lateral ventricles. **B**. (Case 1) Tufted astrocytes and tau positive neurons in the caudate nucleus (tau stain (AT8), magnification 200×). **C**. (Case 1) Coiled bodies in the parietal white matter (tau stain (AT8), magnification 400×). **D**. (Case 1) Tufted astrocytes and tau positive neurons in the parietal cortex (tau stain (AT8), magnification 200×). **E**. (Case 3) Lewy bodies in the substantia nigra (H&E, magnification 400×). **F**. (Case 3) Substantia nigra, Lewy bodies and Lewy neuritis (alpha-synuclein, magnification 200×).

This 68-year-old woman developed balance impairment followed by falls, mostly forward, urinary incontinence, and intermittently slurred speech over a 2-year period. Head CT demonstrated mild to moderate hydrocephalus (Figure 1A), prompting referral for VPS placement. On neurological examination, she exhibited a tremorless parkinsonian phenotype with cognitive impairment (MMSE = 19/30) associated with echolalia, growling dysarthria, and hypomimia, prompting a revised clinical diagnosis of PSP. By three years from symptom onset, she developed square-wave jerks, slow saccades, and supranuclear vertical gaze palsy (Additional file 1: Video S1). Donepezil, rivastigmine, and memantine trials provided no benefit. She died after six years from symptom onset. Neuropathological studies confirmed the diagnosis of PSP with concurrent AD neuropathologic changes (Braak stage II; CERAD score A) and mild associated arteriolosclerosis and atherosclerosis (Figure 2A-D).

### Case 2

This 79-year-old man developed difficulty with “complex” leg movements, such as those required when walking across a railroad or stepping on a train wagon. Brain MRI obtained within one year was interpreted as mild hydrocephalus (Figure 1B), prompting a referral for was consideration of VPS placement. His examination showed parkinsonism with marked gait and postural impairment, upgaze restriction, and stimulus-sensitive axial myoclonus (Additional file 2: Video S2), as well as mild dysexecutive impairment (MMSE = 28/30; Frontal Assessment Battery = 14/18; Montreal Cognitive Assessment = 25/30). His revised clinical diagnosis was PSP and VPS placement was discouraged. Death occurred 10 years after symptom onset. Post-mortem studies confirmed the diagnosis of PSP with associated low-grade AD neuropathology (Braak stage II; CERAD score A) and brainstem Lewy bodies.

### Case 3

This 73-year-old man initially exhibited falls, visual hallucinations, irritability and combative behavior. Within two months he was unable to ambulate independently and had noticeable memory loss. Brain MRI taken at 3 months from symptom onset demonstrated moderate ventriculomegaly without overt atrophy in the apical cuts, suggesting the diagnosis of NPH (Figure 1C). However, in short sequence he developed dysarthria evolving to anarthria, severe dysphagia, and needed assistance for all activities of daily living. At 5 months, his examination showed retrocollis with axial-predominant rigidity and ophthalmoplegia, taking place at one of his “bad spells” (Additional file 3: Video S3). Cognitive assessment was impossible given marked dysarthria. Rivastigmine provided no benefits but donepezil eliminated his visual hallucinations. A rapidly progressive form of PSP was suspected and shunting was discouraged. Despite the early rapid progression, death from aspiration pneumonia occurred six years after symptom onset. Post-mortem neuropathology unexpectedly revealed DLB (neocortical lewy body type, Braak stage V; high likelihood) (Figure 2E-F) with mild to moderate arteriolosclerosis and mild AD neuropathology (Braak stage II; CERAD score A).

### Case 4

This 72-year-old man with progressive gait and balance impairment underwent a brain MRI, which upon presence of hydrocephalus, hyperdynamic fluid signal in the third ventricle, and entrapped sulci in several cortical areas (Figure 1D) prompted a referral to our center. Initial examination 18 months after symptom onset demonstrated mild non-fluent aphasia, visuospatial dysfunction, and delayed recall (MMSE = 24; Montreal Cognitive Assessment = 13/30] in the setting of wide based, unsteady gait. Mild impairment of upgaze with absent vertical optokinetic responses was present at the initial examination (Additional file 4: Video S4). External lumbar drainage provided benefits in gait and urinary function. VPS placement, however, was followed by improvements in gait and urinary function for about 6 months, followed by deterioration of his overall parkinsonism and cognitive function. He developed frontal release signs and disinhibition, with worsening of dysexecutive dementia, mild facial dystonia, greater oculomotor dysfunction (impairment of upgaze, slowed downgaze, square-wave jerks), and marked postural impairment with backward falls. He died from aspiration pneumonia six years from onset of symptoms. Suspected PSP was confirmed on postmortem studies, which also revealed unusually marked and widespread occipital cortical involvement.

## Discussion

There are two major shortcomings in the appraisal of NPH as an entity, namely the absence of clinical and pathological biomarkers and the reliance on short-term response to drainage for its definition [2], which often does not “hold water” in the long term [1]. Indeed, the longer the follow up, the lower the frequency of response, and the higher the diagnostic revision rate [1]. Important lessons derived from the suspected NPH patients presented here were that cognitive impairment predated or occurred concurrently with gait dysfunction, and impairment of postural reflexes was universal. This is in keeping with the observation that postural instability at the initial evaluation of a suspected NPH patient predicts the absence of sustained improvement at 3 years from VPS placement [1].

Besides early impairment of postural reflexes, our patients had early cognitive impairment, either predating or occurring concurrently with gait impairment. Cognitive impairment is a regrettable component of the “classic” NPH triad. Of the 8 cognitively impaired VPS-treated patients in the Mayo Clinic series, only 1 continued to benefit at 3 years [1]. This implies that by the time a patient with hydrocephalus develops cognitive impairment, the odds of shunt responsiveness are substantially low, and diagnostic considerations other than NPH are warranted. Patients may have AD if cognitive impairment predominates [3] and PSP or DLB if parkinsonism is an important clinical manifestation. In the former situation, AD biomarkers may be particularly helpful in discriminating AD from true NPH. In a sample of 37 prospectively followed VPS-treated “NPH” patients, poor sustained response was predicted by the presence of amyloid β plaques, neuritic plaques, and/or neurofibrillary tangles observed in cortical biopsies taken at the time of shunt insertion [3]. Cortical biopsies identified amyloid-β aggregates in over half of presumed NPH patients who had initial response to VPS but eventual progression of dementia [4]. Similarly, 8 out of 9 patients with clinically diagnosed NPH that came to autopsy at the Sun Health Research Institute within a 12-year period, demonstrated AD pathology (one also with DLB) and the other met pathologic criteria for PSP [5].

A major pitfall of the interpretation of these data is to assume that these patients had “co-occurrence” of AD and NPH [5, 6], rather than the hydrocephalic presentation of a single, neurodegenerative disorder. This potential misinterpretation is giving rise to reports of NPH patients “evolving” to AD [7], without considering the possibility that NPH may have been an early clinical misdiagnosis. Although the postulation of an “AD-NPH syndrome” [8, 9] has already led to a negative clinical trial [10], clinicians continue to offer VPS to patients with hydrocephalus in the setting of dementia, because it is part of the “classic triad” (conversely, some neurosurgeons may be reluctant to shunt if dementia is not present in patients whose hydrocephalus may “only” be associated with gait impairment).

PSP appears to be the most common etiology of suspected NPH with a parkinsonian phenotype (Table 1). It was present in 3 of 4 such cases from the Queen Square Brain Bank, none of whom had, at presentation, any oculomotor dysfunction [11]. However, postural instability and mild dysexecutive impairment were present in all. VPS placement in three resulted in short-term benefits but subsequent deterioration ensued within one year. The early Toronto experience showed that 3 of 5 NPH patients with hydrocephalus and early response to VPS evolved into a parkinsonian phenotype confirmed to represent PSP in two (one with pathology confirmation) and DLB in another [12].

**Table 1:** **NPH-like presentations for pathology-confirmed PSP and DLB cases: summary of the published literature**

Case	Age, gender	Disease duration at presentation	Initial clinical features	Late clinical features	Response to VPS	Pathology
1 [1]	82, F*	1.1 years	GD, CI, UI, postural instability	N/A	Transient improvement in GD/UI; worse at 3 years	PSP
2 [5]	77, M^♣^	N/A	GD, CI, UI	N/A	VPS placed, outcome data not available	PSP
3 [11]	78, F	2 years	GD, CI, UI, falls, generalized bradykinesia, dysarthria	Restriction of vertical eye movements, hypophonia, frontal release signs	Not placed	PSP
4 [11]	68, M	4 months	GD, CI, UI, gait ignition failure, falls, hypomimia, micrographia	Restriction of vertical gaze, slow saccades, dysarthria, gait freezing	Transient improvement but progressive dementia	PSP
5 [11]	66, F	2 years	GD, CI, falls, micrographia	Restriction of vertical gaze, slow saccades, dysarthria, dysphagia, gait freezing, dementia	Transient improvement in GD/CI but progressive dementia	PSP
6 [12]	69, M	N/A	GD, CI, UI	VSGP, akinesia, rigidity	6-month “marked” improvement in GD, CI, and UI	PSP
7 [1]	66, F^♦^	3.1 years	GD, CI, UI, postural instability	“Clinical diagnosis of DLB”	Some improvement in GD at 3–6 months; no improvement at 3 years	DLB
8 [5]	80, M^♠^	N/A	GD, CI	N/A	VPS placed, outcome data not available	DLB
9 [7]	87, F	2 years	GD, CI, UI	Postural instability, falls, rare visual hallucinations	Initial marked improvement in GD/CI – No follow-up data.	DLB

GD: gait disturbance; CI: cognitive impairment; UI: urinary incontinence; VSGP: vertical supranuclear gaze palsy. *: VPS placed at 82 (no age at presentation given); ♣: Death at 77 (no age at presentation given); ♦: VPS placed at 66 (no age at presentation given); ♠: Death at 80 (no age at presentation given).

Our cases and the review of the literature highlight several key clinically relevant messages: 1) early cognitive impairment is the most important “cognitive red flag” of the NPH triad and predicts an alternative diagnosis; 2) early impairment of postural reflexes, with or without falls, is the most common “motor red flag” of the NPH triad, and should suggest PSP or DLB; 3) visual hallucinations are a strong clinical biomarker of synucleinopathies [13] and their presence in the setting of hydrocephalus tilts the diagnostic yield toward DLB; and 4) a “honeymoon” period may occur after shunting in patients with PSP but further progression would be expected thereafter. To the extent that transient symptomatic relief in the setting of a neurodegenerative disorder may be preferable to no relief at all, the approach to a hydrocephalic patient with “red flags” may still include fluid diversion, but patients and families need to be counseled about the anticipated short-term gains.

In sum, hydrocephalus in the setting of postural impairment and/or oculomotor abnormalities most probably represents PSP or DLB. Hallucinations may be the only early manifestation of the hydrocephalic presentation of DLB. An initial promising response to VPS placement does not exclude the possibility of an underlying neurodegenerative disease. Furthermore, a fully formed “classic” triad of gait impairment, urinary incontinence, and cognitive impairment appears to be more common in conditions other than NPH. The accumulating evidence suggests that early postural impairment, falls, oculomotor impairment, or hallucinations are inconsistent with the diagnosis of NPH regardless of ventricular size. Recognition of the hydrocephalic presentation of PSP and DLB can avoid unnecessary and potentially harmful shunting procedures.

## Electronic supplementary material

Additional file 1: Video S1: Patient 1 shows growling dysarthria, restriction of upgaze with supranuclear vertical gaze palsy, appendicular dystonia with dysmetria, wide-based, short-stride gait with erect posture, mild retrocollis, and impaired postural reflexes. (MP4 11 MB)

Additional file 2: Video S2: Patient 2 demonstrates upgaze restriction with nystagmus but no square-wave jerks or slow saccades. There was marked gait impairment with wide base support and retropulsion on pull test. (MP4 17 MB)

Additional file 3: Video S3: Patient 3 shows marked retrocollis, severe dysarthria and ophthalmoplegia. Patient is videotaped over the course of a “bad spell”, in hindsight interpreted as part of his DLB-associated cognitive fluctuations. (MP4 13 MB)

Additional file 4: Video S4: Patient 4 shows a wide based, unsteady gait. Disappearance of corrective saccades to the optokinetic strip was the early clue as to his subtle saccadic impairment, and predated a later development of supranuclear vertical gaze palsy. (MP4 13 MB)

Below are the links to the authors’ original submitted files for images.

Authors’ original file for figure 1

Authors’ original file for figure 2
