# Supplementary figures and images for: Hydrocephalic Parkinsonism: lessons from normal pressure hydrocephalus mimics
Source: J Clin Mov Disord. 2014 Oct 29;1:2. doi: 10.1186/2054-7072-1-2 (PMC4677733; doi:10.1186/2054-7072-1-2)

## Slide 1
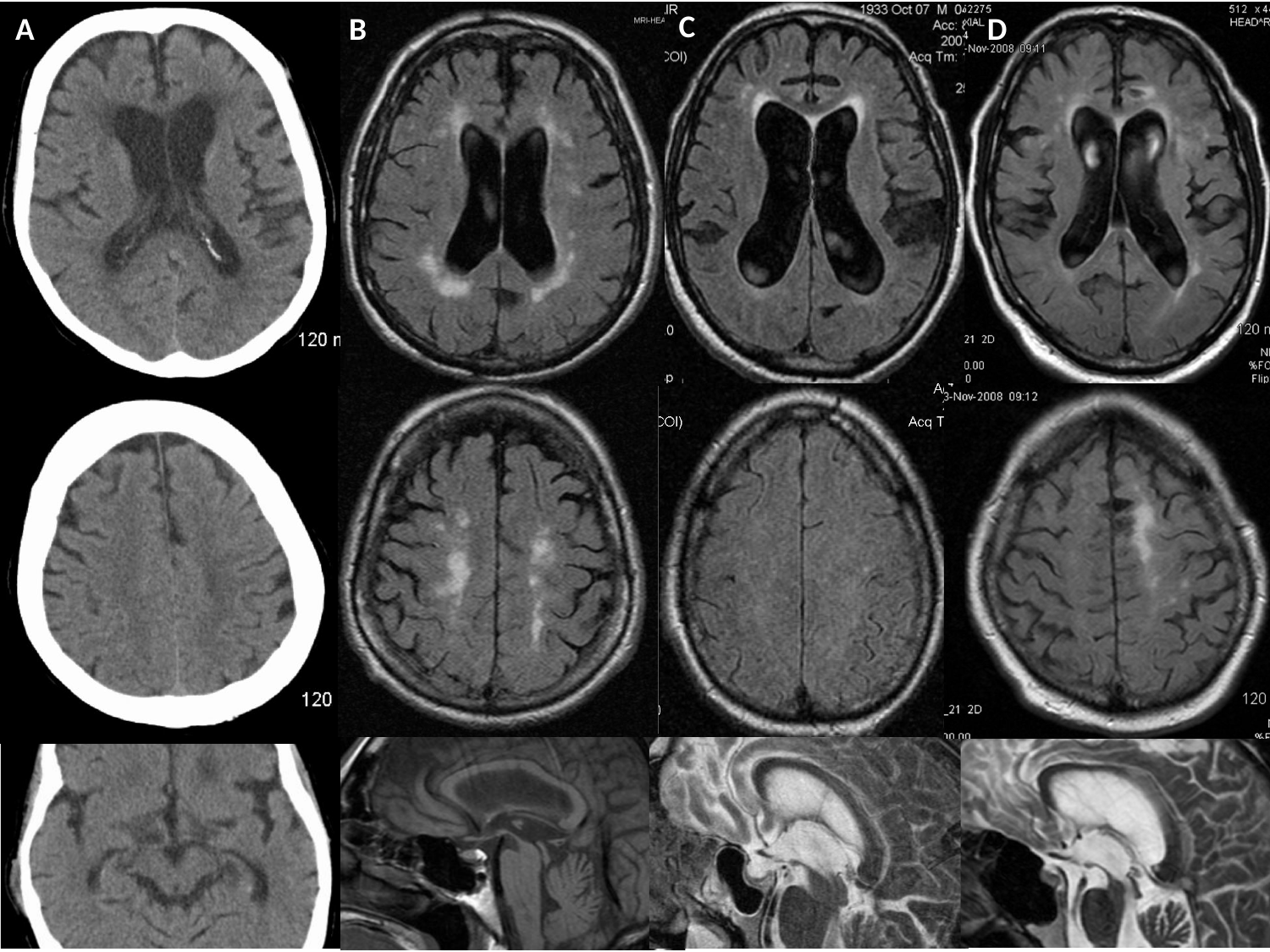

C
A
B
D

Supplement: Supplementary file 5 — Authors’ original file for figure 1 [file 40734_2014_2_MOESM5_ESM.pptx]

## Slide 1
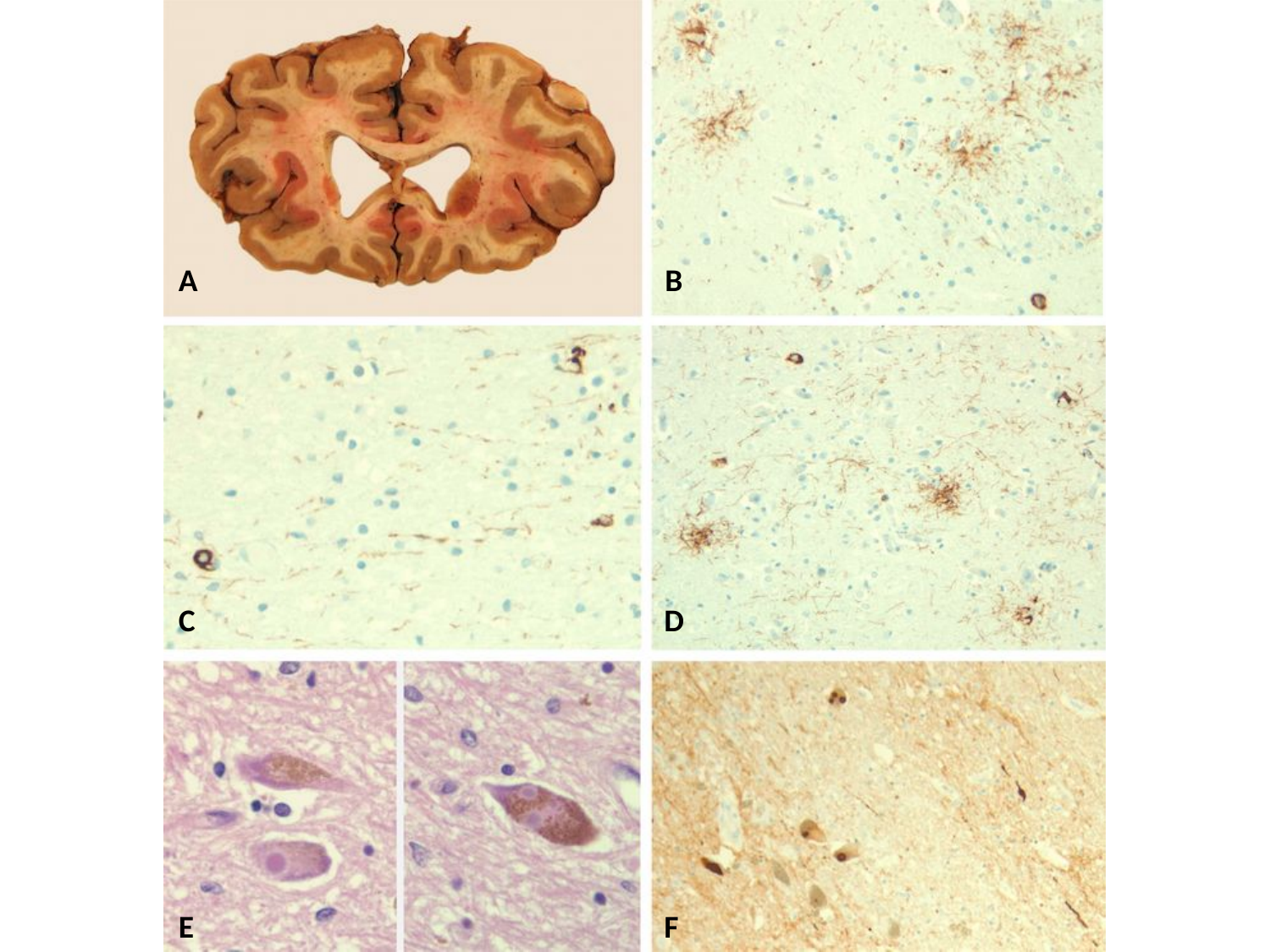

A
B
C
D
E
F

Supplement: Supplementary file 6 — Authors’ original file for figure 2 [file 40734_2014_2_MOESM6_ESM.pptx]
